# Supplementary material for: Quantitative analysis of electroporation-mediated intracellular delivery via bioorthogonal luminescent reaction
Source: Commun Chem. 2024 Aug 15;7:181. doi: 10.1038/s42004-024-01266-4 (PMC11327378; doi:10.1038/s42004-024-01266-4)
Supplement: Supplementary file 2 — Supplementary Information [file 42004_2024_1266_MOESM2_ESM.docx]

**Supplementary Information**

**Quantitative analysis of electroporation-mediated intracellular delivery via bioorthogonal luminescent reaction**

Shiqi Wang^1*^, Mariia V. Shcherbii^2^, Sami-Pekka Hirvonen^3^, Gudrun Silvennoinen^3^, Mirkka Sarparanta^3^, and Hélder A. Santos^1,4^

^1^Drug Research Program, Division of Pharmaceutical Chemistry and Technology, Faculty of Pharmacy, University of Helsinki, FI-00014 Helsinki, Finland.

^2^Institute of Biotechnology, University of Helsinki, FI-00014 Helsinki, Finland.

^3^Department of Chemistry, Faculty of Science, University of Helsinki, FI-00014 Helsinki, Finland

^4^Department of Biomaterials and Biomedical Technology, The Personalized Medicine Research Institute (PRECISION), University Medical Center Groningen, University of Groningen, 9713 AV Groningen, The Netherlands.

^*^ (S.W.) Email: [shiqi.wang@helsinki.fi](mailto:shiqi.wang@helsinki.fi)

**Figure S1.** The cell viability (measured by intracellular ATP amount) after electroporation, normalized by negative control (cells without electroporation). Data are presented as the mean ± s.d. (n = 4). n.s., not significant, ** p < 0.05*, from Student’s *t*-test (unpaired and two-tailed).

**Figure S2.** Real-time luminescence signal of electroporated cells with Dcystine, Lcystine, and without any reagent (Ctrl). The signal was recorded right after the addition of NCBT. Data are presented as the mean ± s.d. (n = 3). The error bars are presented as filled areas.

**Figure S3.** Up, the synthetic scheme of cysteine-modified dextran (Dex-Lcys and Dex-Dcys). Down, the ^1^H-NMR spectra of amino-dextran (Dex-amine), SPDP modified dextran (Dex-SPDP) and Dex-Dcys, in *DMSO-d_6_*. The peaks from 7.0 to 8.6 ppm in the enlarged image are designated to the pyridine ring of SPDP.

**Figure S4.** Up, the synthetic scheme of Dcys-labelled dextran via non-cleavable thiol-maleimide reaction (Dex-NC). Down, the ^1^H-NMR spectra of amino-dextran (Dex-amine), maleimide modified dextran (Dex-mal) and Dex-NC, in *DMSO-d_6_*. The sharp peak at 7 ppm is designated to maleimide.

**Figure S5.** The SEC curves of Dex-Dcys, Dex-Lcys and Dex-NC from refractive index (RI) detector and the fluorescence detector.

**Figure S6.** Mean fluorescence intensity (MFI) of the electroporated cells with Alexa647 labeled dextran polymers. NE means cells incubated with Alexa647 labeled dextran polymers but without electroporation. Ctrl means cells without Alexa647 labeled dextran polymers exposure. Data are presented as the mean ± s.d. (n = 3).

**
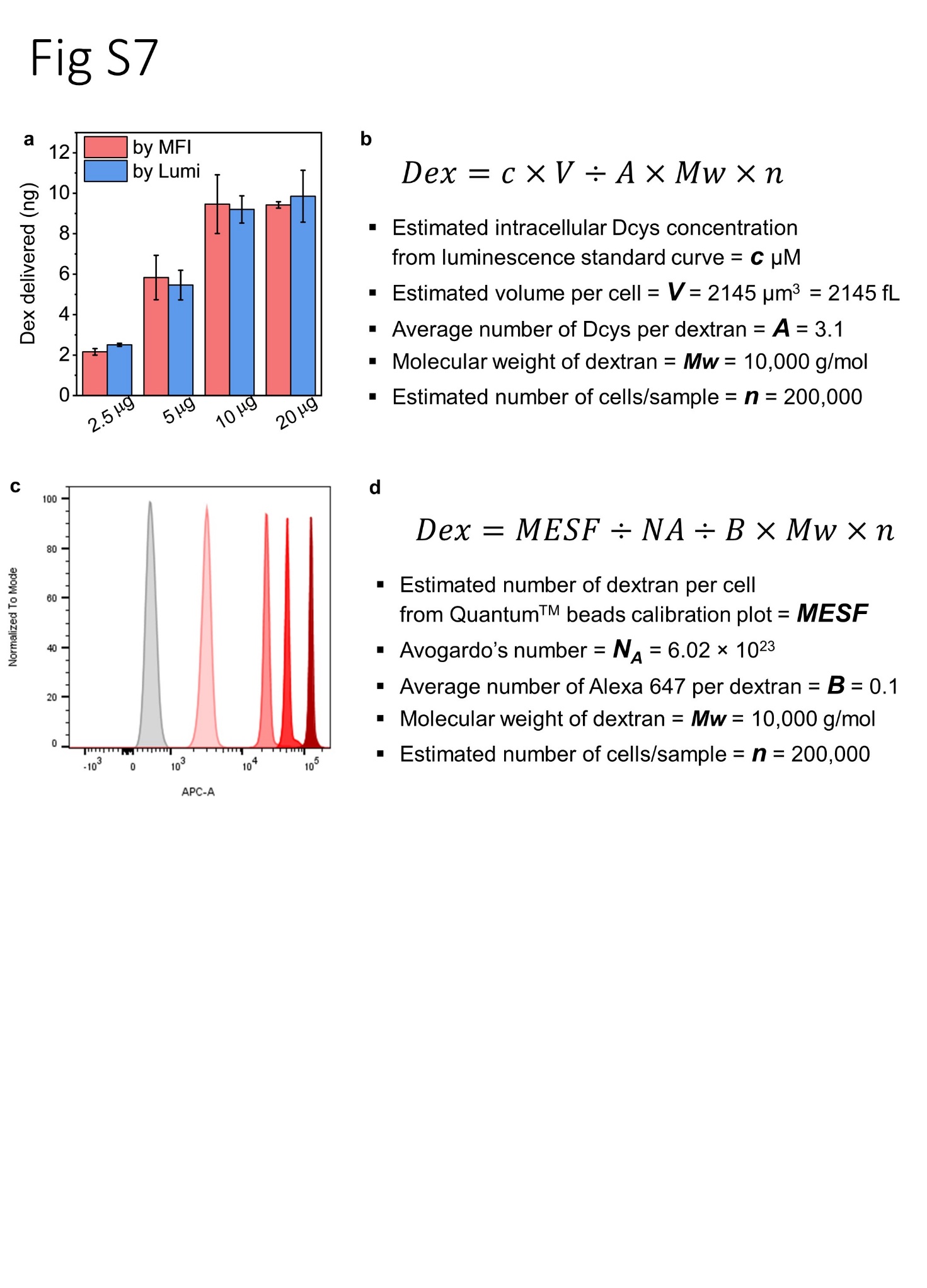
**

**Figure S7. (a)** The estimated total intracellular Dex-Dcys amount per sample (200,000 cells) using quantitative flow cytometry method (By MFI, red) and BioLure assay (By Lumi, blue). Data are presented as the mean ± s.d. (n = 3). **(b)** The equation and key parameters used for BioLure assay calculation. **(c)** The original flow cytometry data of Alexa647 fluorescence calibration Quantum™ beads. **(d)** The equation and key parameters used for quantitative flow cytometry calculation.

**
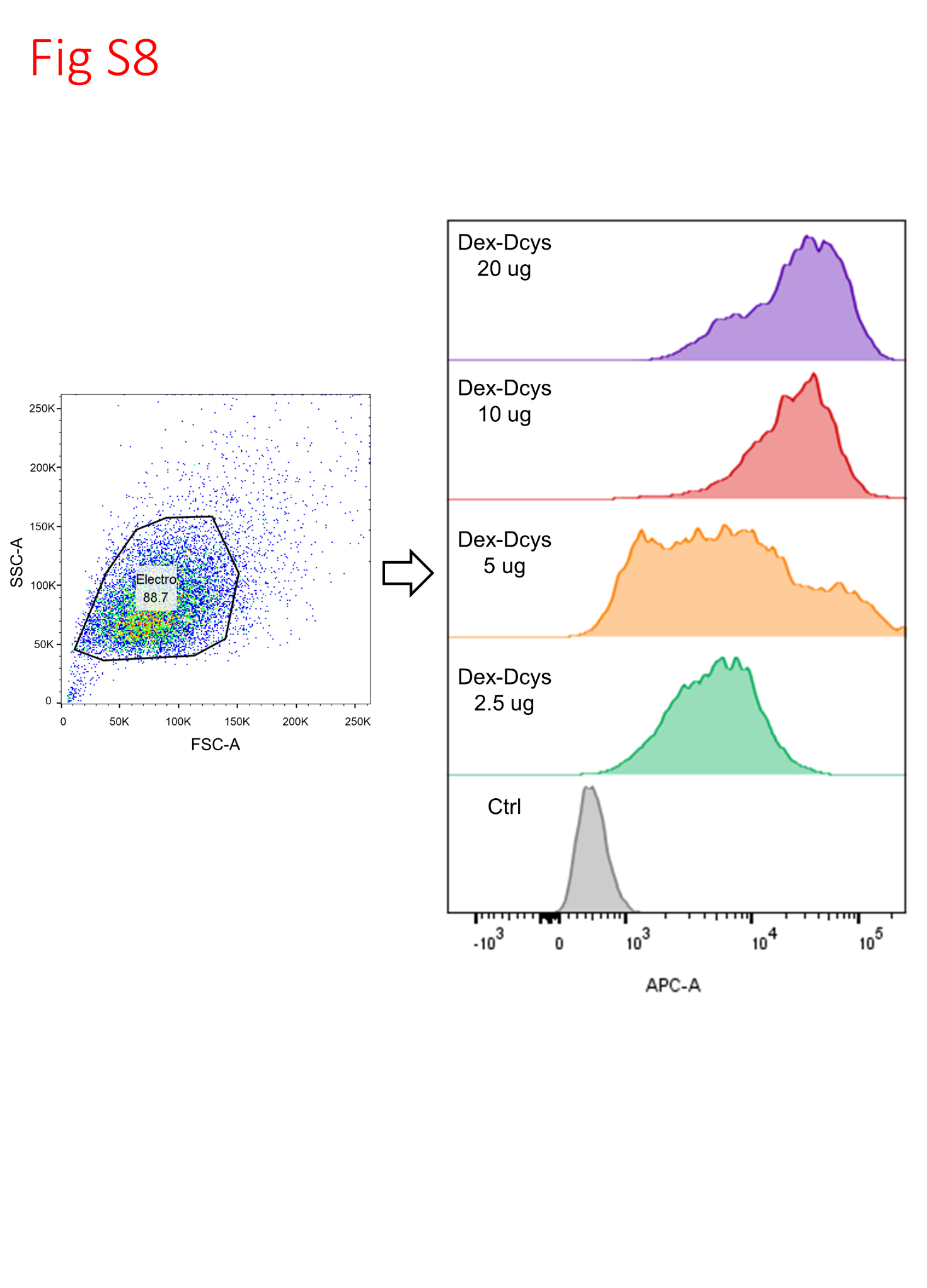
**

**Figure S8.** The flow cytometry gating strategy and the histogram of cells electroporated with different amount of Dex-Dcys (2.5 to 20 µg). Ctrl means electroporated cells without any polymer.


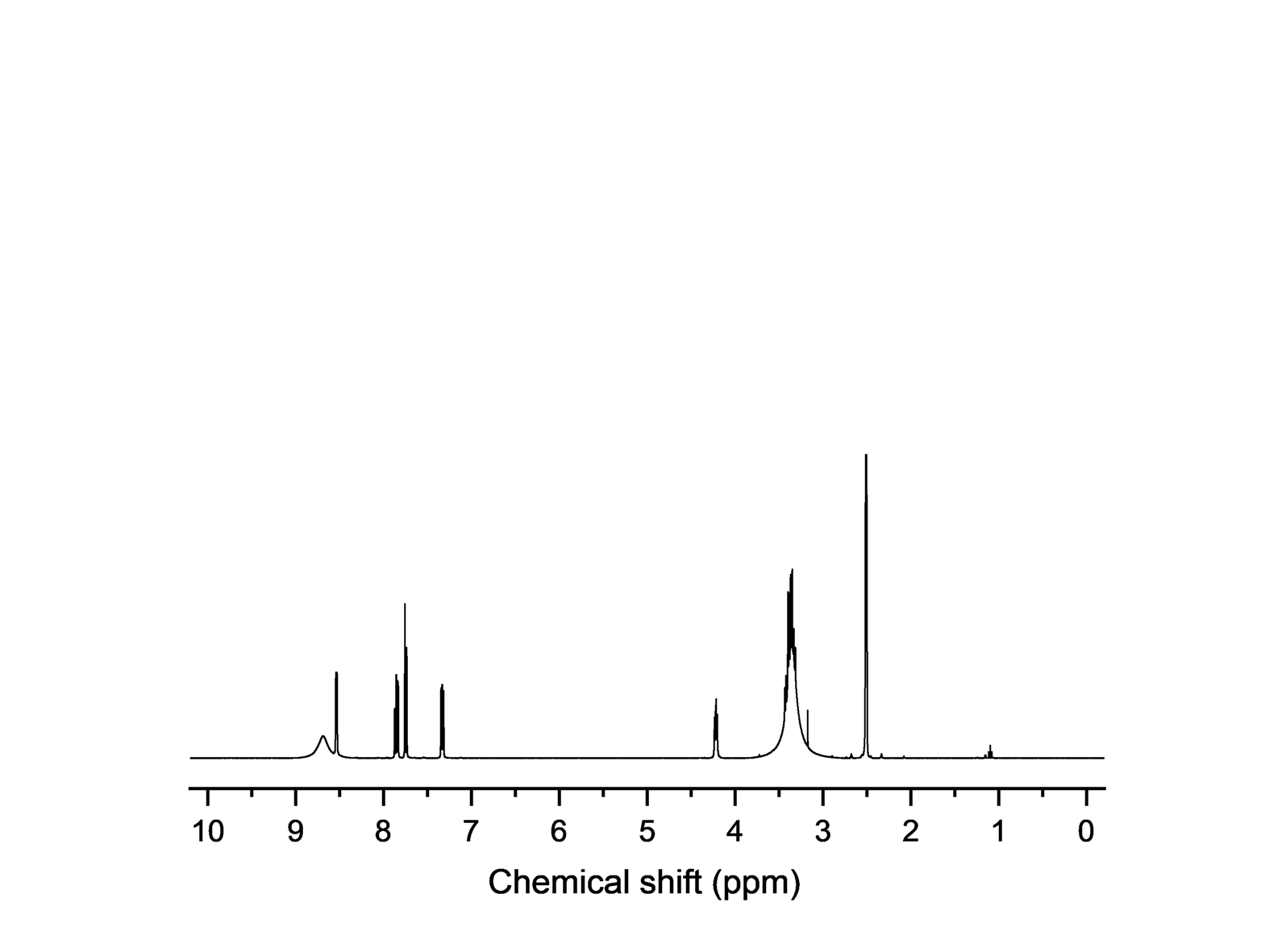


**a**

**b**

**c**

*DMSO-d_6_*

**a**

**b**

**c**

**Figure S9.** The ^1^H-NMR spectra of Py-Dcys with proton designation, in *DMSO-d_6_*.

**
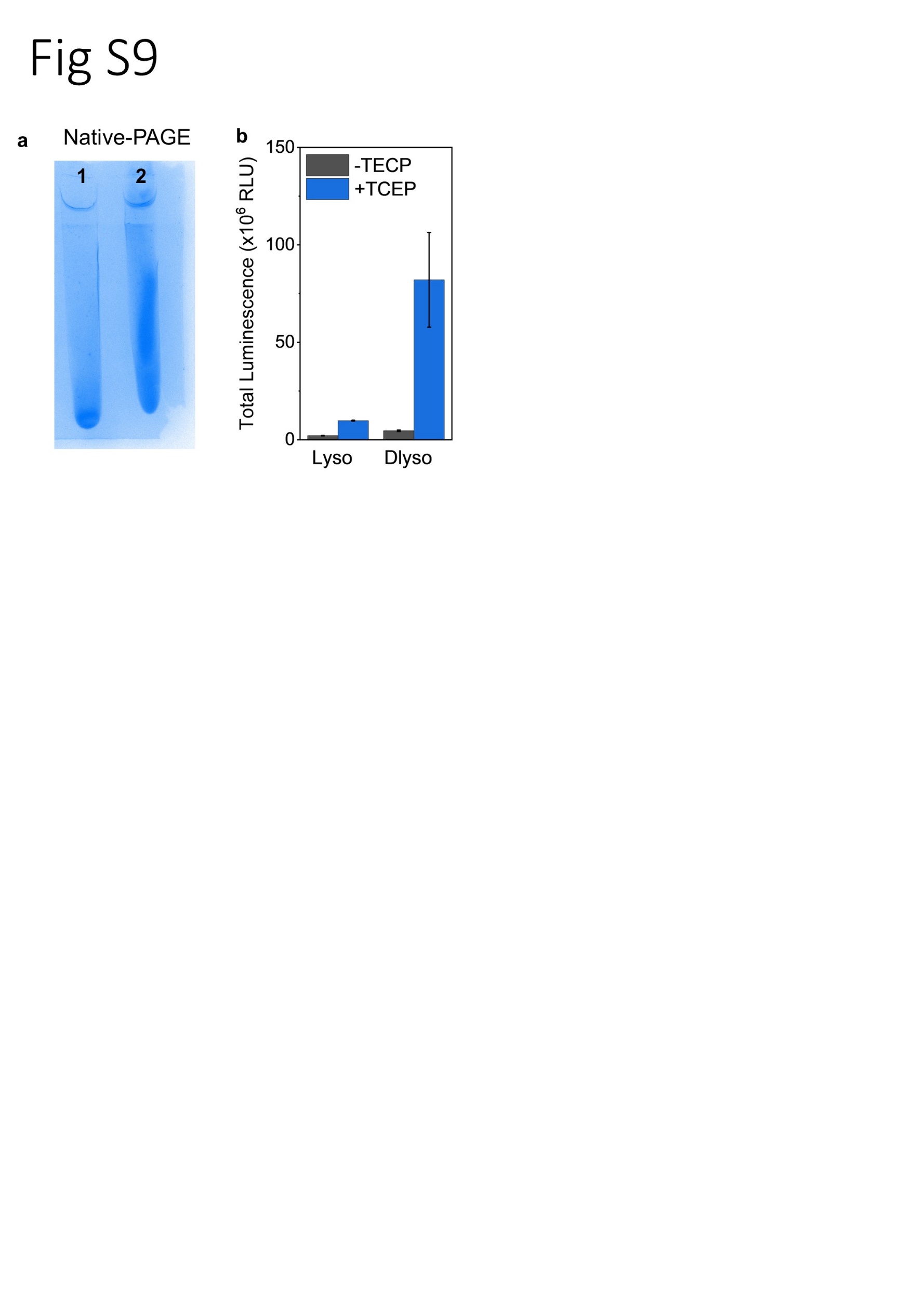
**

**Figure S10. (a)** Image of native-PAGE gel of lysozyme (Lane 1) and Dlyso (Lane 2). **(b)** Total luminescence output (from 0 to 30 min) of the extracted proteins after bioorthogonal reaction in the reaction buffer. Lyso means lysozyme without modification. Dlyso means lysozyme with Dcys tag. TCEP is added to reduce Dcys from Dlyso. Data are presented as the mean ± s.d. (n = 4).


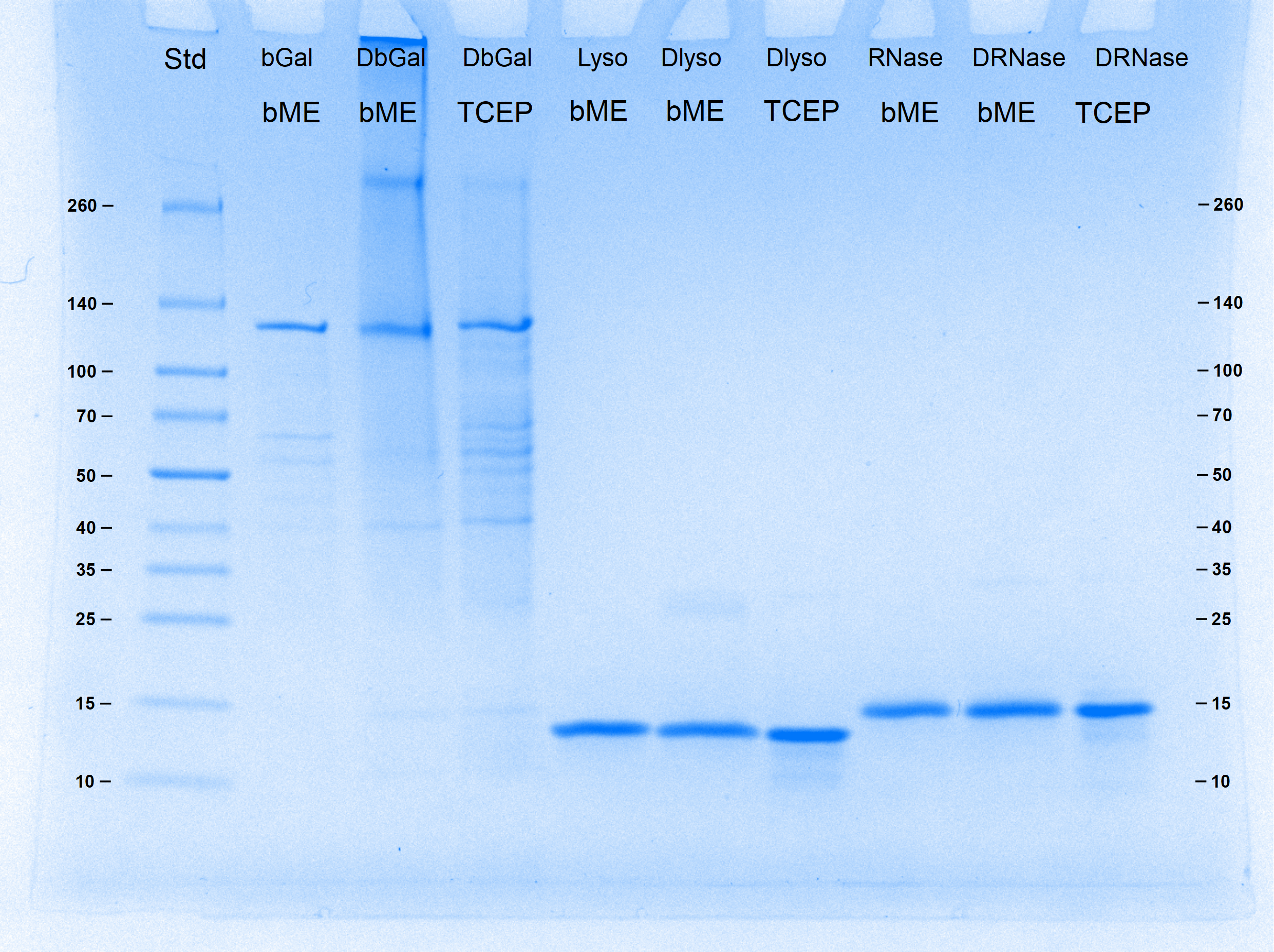


**Figure S11.** Image of reducing SDS-PAGE gel of Dcys modified proteins (1 µg per lane). From Left to Right: Lane 1, protein standards. Lane 2, bGal reduced by β-mercaptoethanol. Lane 3, DbGal reduced by β-mercaptoethanol. Lane 4, DbGal reduced by TCEP. Lane 5, lysozyme reduced by β-mercaptoethanol. Lane 6, Dlyso reduced by β-mercaptoethanol. Lane 7, Dlyso reduced by TCEP. Lane 8, RNase reduced by β-mercaptoethanol. Lane 9, DRNase reduced by β-mercaptoethanol. Lane 10, DRNase reduced by TCEP.


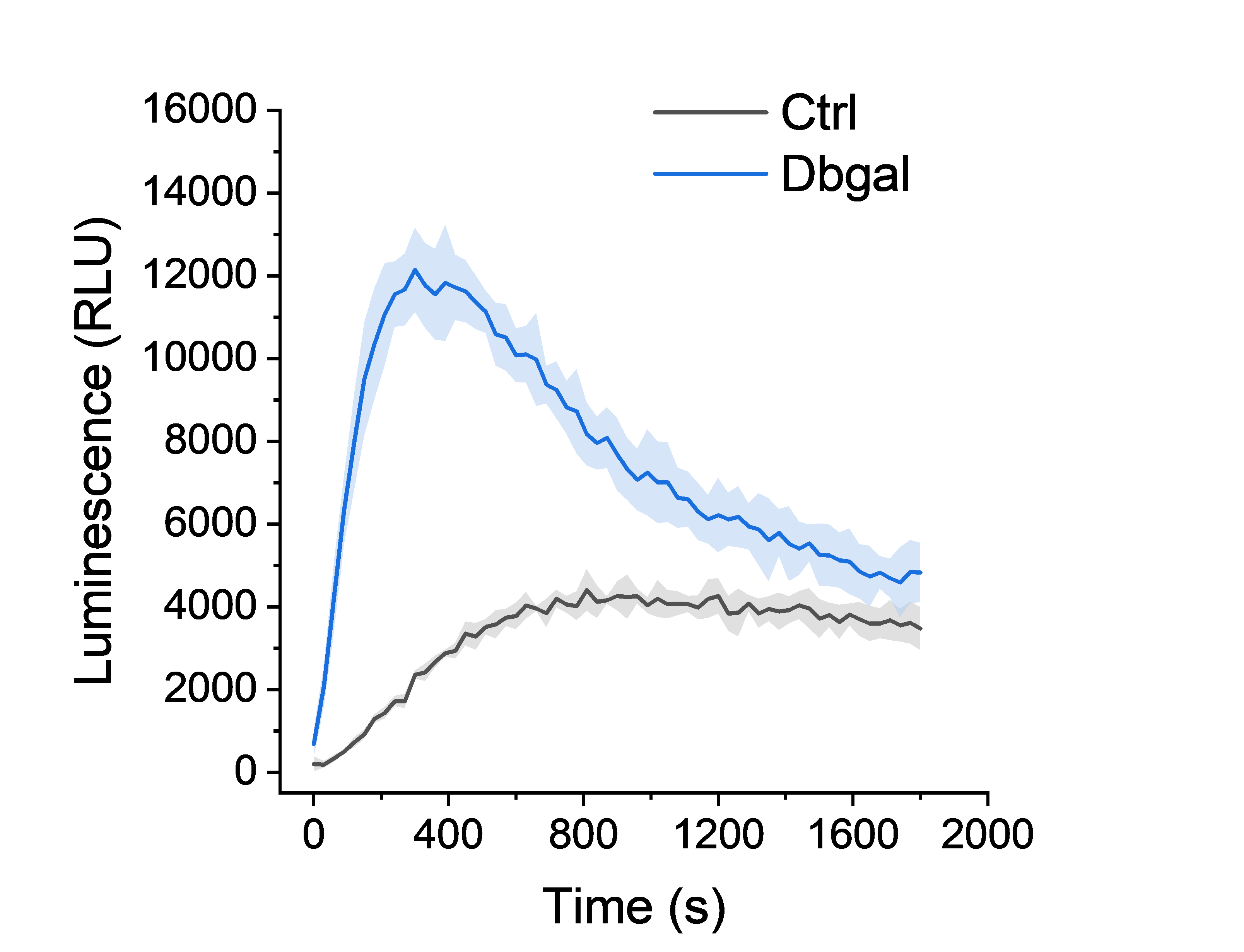


**Figure S12.** Real-time luminescence signal of electroporated A375-Fluc-eGFP cells with DbGal and without DbGal (Ctrl). Data are presented as the mean ± s.d. (filled area, n = 3).

**
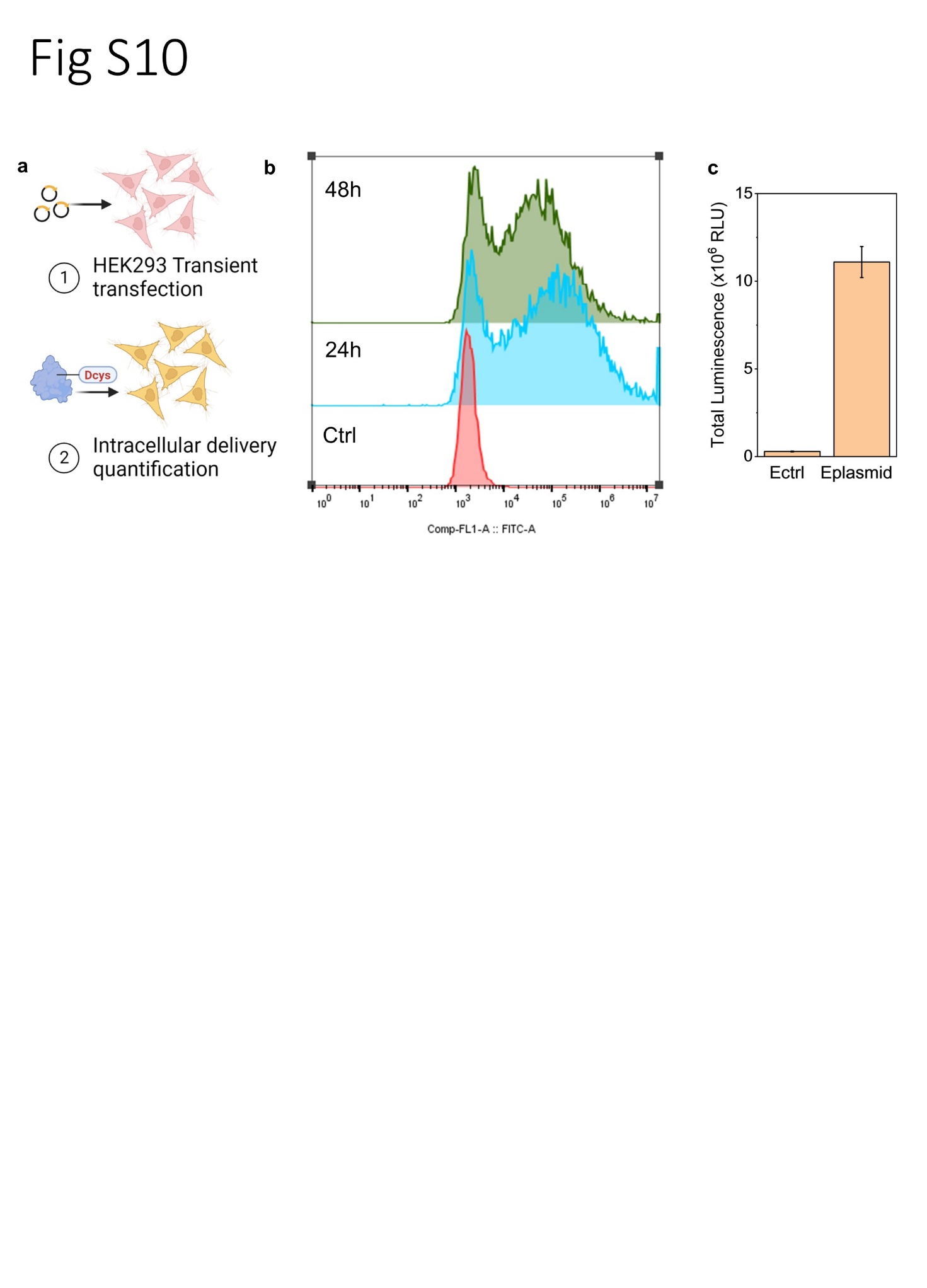
**

**Figure S13. (a)** Scheme of the transiently transfected HEK293 cells used in the BioLure assay. Created with BioRender.com. **(b)** The flow cytometry results of HEK293 cells eGFP expression after 24 h and 48 h electroporation. Ctrl, cells electroporated without plasmids. **(c)** The luminescence of transfected HEK293 cells after the addition of luciferase substrate (D-amLu). Ectrl, cells electroporated without plasmids. Data are presented as the mean ± s.d. (n = 2).

**
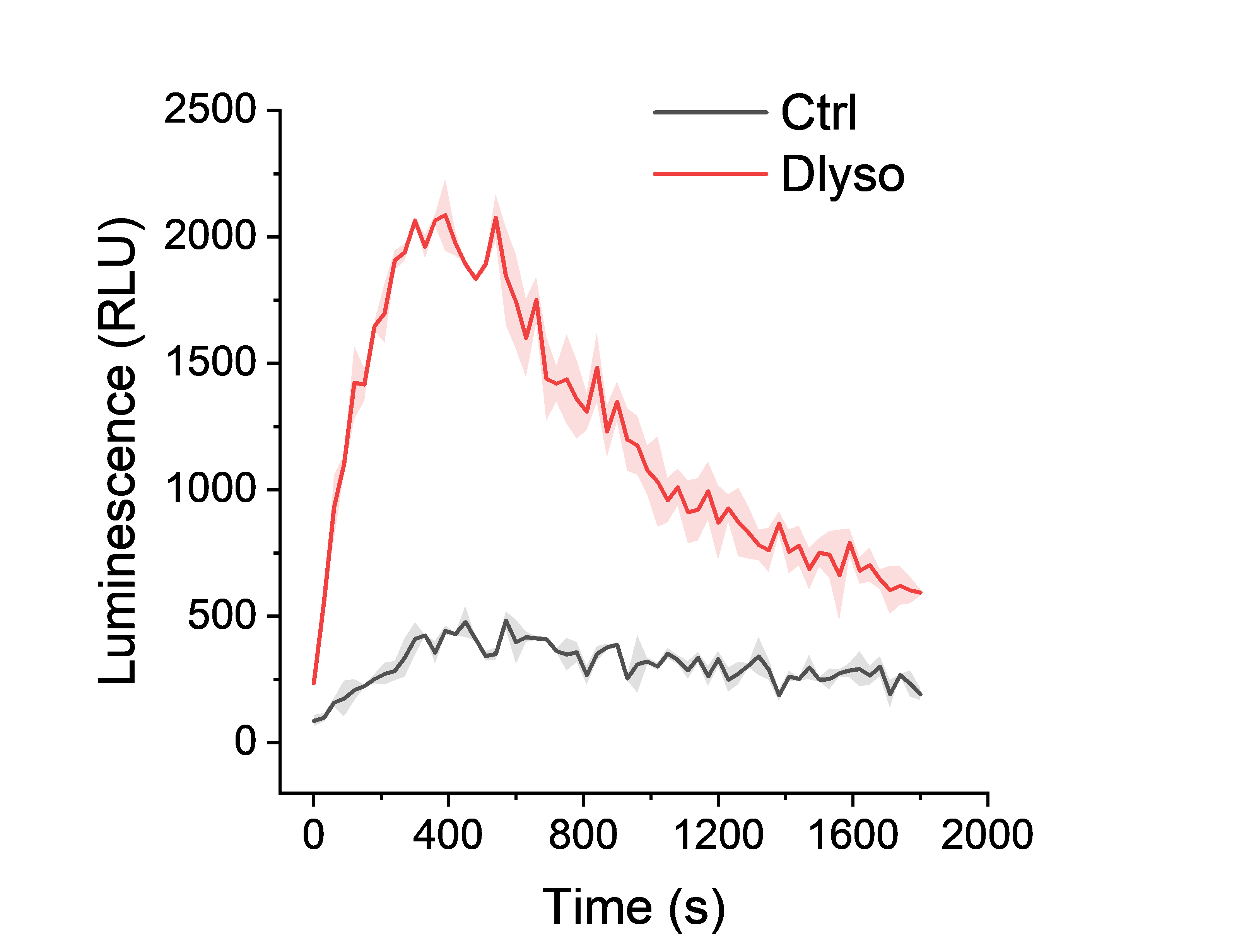
**

**Figure S14.** Real-time luminescence signal of electroporated luciferase-expressing HEK293 cells with Dlyso and control. Data are presented as the mean ± s.d. (filled area, n = 3).

**
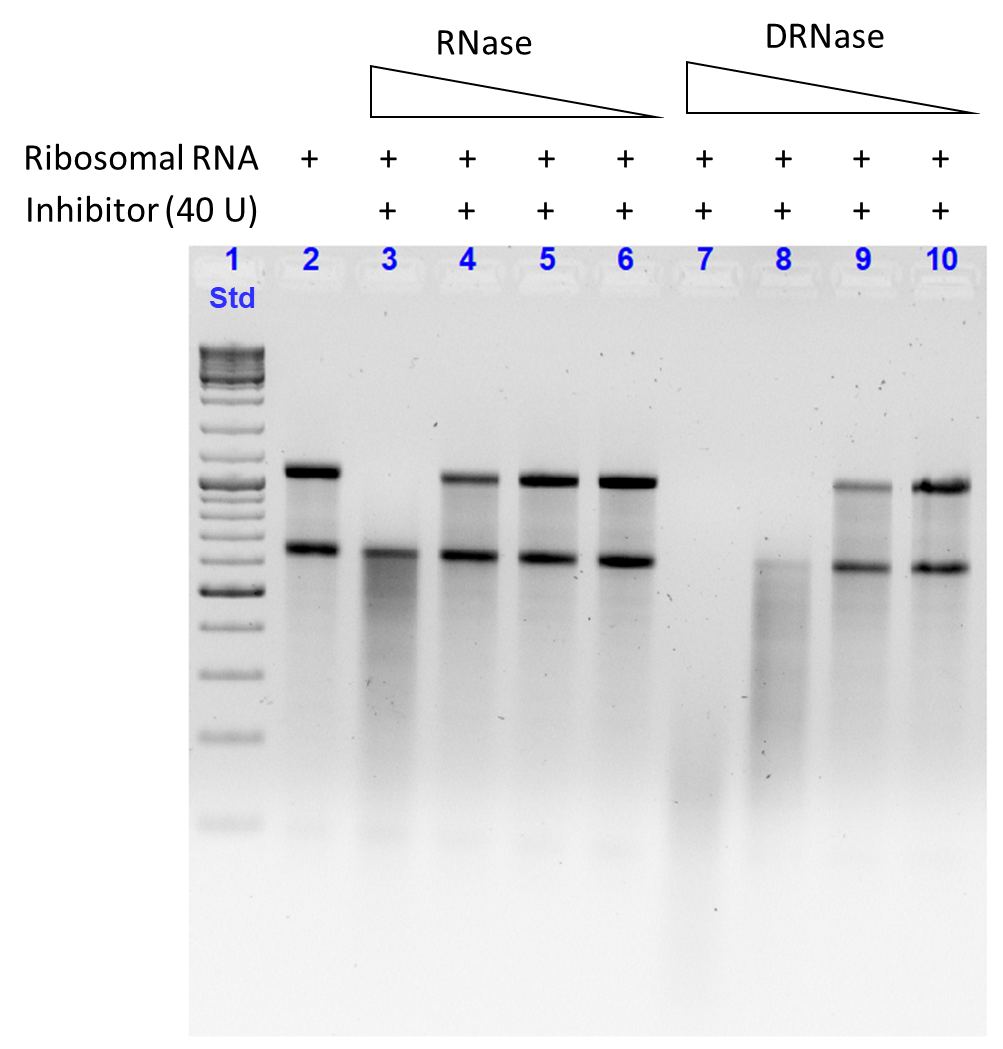
**

**Figure S15.** The agarose gel of ribosomal RNA degradation by RNase and DRNase, Lane 1, ladder. Lane 2, RNA control (1 µg) without any ribonuclease. Lane 3-6, RNase concentration gradient: 40 ng, 4 ng, 0.4 ng and 0.04 ng per sample. Lane 7-10 the same concentration gradient of DRNase. Inhibitor amount is fixed at 40 U per sample.

**
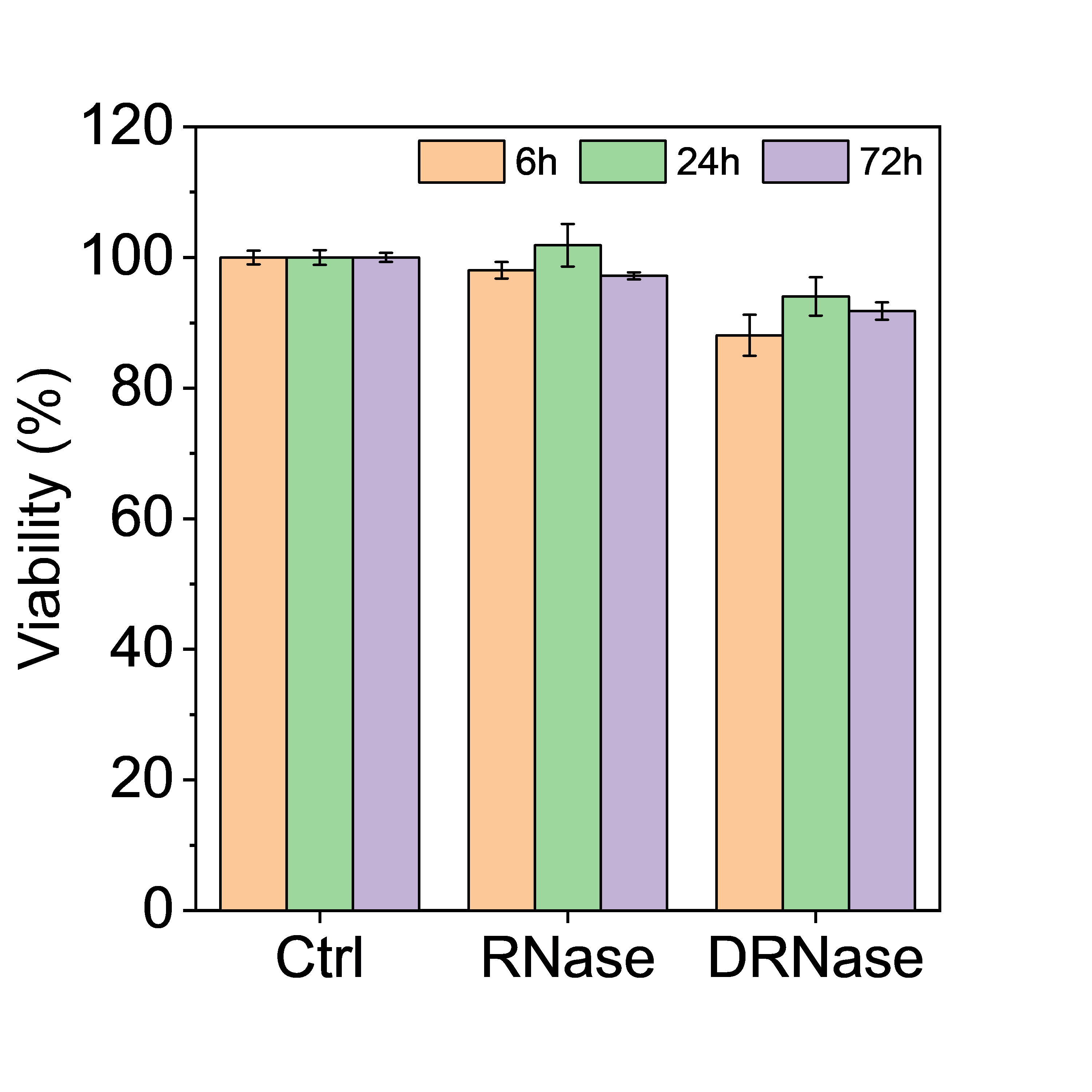
**

**Figure S16.** The cell viability after 6, 24, and 72 h incubation with RNase or DRNase in the culture medium without electroporation. Data are presented as the mean ± s.d. (n = 4).

**Table S1.** The elemental analysis results of dextran derivative polymers (N =2).

| Polymer | N % | C % | H % | S % |
| --- | --- | --- | --- | --- |
| Dex-amine | 0.97 ± 0.00 | 41.18 ± 0.14 | 6.64 ± 0.04 | 0.05 ± 0.02 |
| Dex-Dcys | 1.29 ± 0.01 | 40.59 ± 0.01 | 6.57 ± 0.04 | 1.66 ± 0.04 |
| Dex-Lcys | 1.20 ± 0.04 | 40.46 ± 0.36 | 6.39 ± 0.10 | 1.25 ± 0.01 |
| Dex-NC | 1.79 ± 0.03 | 41.35 ± 0.17 | 6.52 ± 0.02 | 0.55 ± 0.01 |
