# Supplementary material for: Quantitative analysis of electroporation-mediated intracellular delivery via bioorthogonal luminescent reaction
Source: Commun Chem. 2024 Aug 15;7:181. doi: 10.1038/s42004-024-01266-4 (PMC11327378; doi:10.1038/s42004-024-01266-4)
Supplement: Supplementary file 3 — Reporting Summary [file 42004_2024_1266_MOESM3_ESM.pdf]

nature portfolio

Corresponding author(s): Shiqi Wang

Last updated by author(s): Jul 24, 2024

Reporting Summary

Nature Portfolio wishes to improve the reproducibility of the work that we publish. This form provides structure for consistency and transparency in reporting. For further information on Nature Portfolio policies, see our Editorial Policies and the Editorial Policy Checklist.

Statistics

For all statistical analyses, confirm that the following items are present in the figure legend, table legend, main text, or Methods section.

n/a	Confirmed	
☐	☒	The exact sample size (*n*) for each experimental group/condition, given as a discrete number and unit of measurement
☐	☒	A statement on whether measurements were taken from distinct samples or whether the same sample was measured repeatedly
☐	☒	The statistical test(s) used AND whether they are one- or two-sided<br>*Only common tests should be described solely by name; describe more complex techniques in the Methods section.*
☒	☐	A description of all covariates tested
☒	☐	A description of any assumptions or corrections, such as tests of normality and adjustment for multiple comparisons
☐	☒	A full description of the statistical parameters including central tendency (e.g. means) or other basic estimates (e.g. regression coefficient) AND variation (e.g. standard deviation) or associated estimates of uncertainty (e.g. confidence intervals)
☐	☒	For null hypothesis testing, the test statistic (e.g. *F*, *t*, *r*) with confidence intervals, effect sizes, degrees of freedom and *P* value noted<br>*Give P values as exact values whenever suitable.*
☒	☐	For Bayesian analysis, information on the choice of priors and Markov chain Monte Carlo settings
☒	☐	For hierarchical and complex designs, identification of the appropriate level for tests and full reporting of outcomes
☒	☐	Estimates of effect sizes (e.g. Cohen's *d*, Pearson's *r*), indicating how they were calculated

Our web collection on statistics for biologists contains articles on many of the points above.

Software and code

Policy information about availability of computer code

Data collection	Thermo Fisher SkanIt™ Software for Varioskan™ LUX multimode microplate reader. Leica LAS X microscope software for confocal images acquisition. BD FACSDiva™ Software for flow cytometry data collection.
Data analysis	The plate reader data were analyzed by Microsoft Excel. The confocal images were analyzed by Fiji-ImageJ (1.53c). The flow cytometry data was analyzed by FlowJo (v10.4). The NMR data were analyzed by Mnova. The protein gel images were analyzed by Image Lab (6.0). The graphs were plotted by Origin (2024).

For manuscripts utilizing custom algorithms or software that are central to the research but not yet described in published literature, software must be made available to editors and reviewers. We strongly encourage code deposition in a community repository (e.g. GitHub). See the Nature Portfolio guidelines for submitting code & software for further information.

Data

Policy information about availability of data

All manuscripts must include a data availability statement. This statement should provide the following information, where applicable:

- Accession codes, unique identifiers, or web links for publicly available datasets
- A description of any restrictions on data availability
- For clinical datasets or third party data, please ensure that the statement adheres to our policy

We include a data availability statement in the manuscript.

Research involving human participants, their data, or biological material

Policy information about studies with human participants or human data. See also policy information about sex, gender (identity/presentation), and sexual orientation and race, ethnicity and racism.

Reporting on sex and gender	NA
Reporting on race, ethnicity, or other socially relevant groupings	NA
Population characteristics	NA
Recruitment	NA
Ethics oversight	NA

Note that full information on the approval of the study protocol must also be provided in the manuscript.

Field-specific reporting

Please select the one below that is the best fit for your research. If you are not sure, read the appropriate sections before making your selection.

☒ Life sciences ☐ Behavioural & social sciences ☐ Ecological, evolutionary & environmental sciences

For a reference copy of the document with all sections, see nature.com/documents/nr-reporting-summary-flat.pdf

Life sciences study design

All studies must disclose on these points even when the disclosure is negative.

Sample size	No statistical methods were used to determine the sample size in the study. The sample sizes are described in each figure legend and in methods section.
Data exclusions	No data were excluded from any of the analyses in this study.
Replication	All experiments were repeated for at least three times and experimental findings were reproducible.
Randomization	NA
Blinding	NA

Reporting for specific materials, systems and methods

We require information from authors about some types of materials, experimental systems and methods used in many studies. Here, indicate whether each material, system or method listed is relevant to your study. If you are not sure if a list item applies to your research, read the appropriate section before selecting a response.

Materials & experimental systems

n/a	Involved in the study	
☒	☐	Antibodies
☐	☒	Eukaryotic cell lines
☒	☐	Palaeontology and archaeology
☒	☐	Animals and other organisms
☒	☐	Clinical data
☒	☐	Dual use research of concern
☒	☐	Plants

Methods

n/a	Involved in the study	
☒	☐	ChIP-seq
☐	☒	Flow cytometry
☒	☐	MRI-based neuroimaging

Eukaryotic cell lines

Policy information about cell lines and Sex and Gender in Research

Cell line source(s)	A375-eGFP-Fluc cells (A375-Fluc-Neo/eGFP-Puro) were purchased from Imanis Life Sciences, USA. HEK-293 cells were

Cell line source(s)	obtained from ATCC.
Authentication	All commercial cell lines were authenticated by the suppliers.
Mycoplasma contamination	All cell lines used in this study were tested negative for mycoplasma.
Commonly misidentified lines (See ICLAC register)	NA

Plants

Seed stocks	NA
Novel plant genotypes	NA
Authentication	NA

Flow Cytometry

Plots

Confirm that:

☒ The axis labels state the marker and fluorochrome used (e.g. CD4-FITC).

☒ The axis scales are clearly visible. Include numbers along axes only for bottom left plot of group (a 'group' is an analysis of identical markers).

☒ All plots are contour plots with outliers or pseudocolor plots.

☒ A numerical value for number of cells or percentage (with statistics) is provided.

Methodology

Sample preparation	Details of sample preparation are provided in Methods section. Cells after electroporation were in suspension and washed twice with PBS buffer before flow cytometry analysis. The Quantum™ Alexa Fluor® 647 Molecules of Soluble Fluorochrome (MESF) beads were used as supplied.
Instrument	BD LSR-II
Software	FlowJo for analyzing flow cytometry samples. Quantum™ Alexa Fluor® 647 Molecules of Soluble Fluorochrome (MESF) beads data were analyzed by the QuickCal® analysis template as instructed by the manufacturer (https://www.bangslabs.com/sites/default/files/imce/docs/PDS%20819%20Web.pdf). BD FACSDiva™ logarithmic regression was selected to fit the data.
Cell population abundance	>5000 cells were presented in the gated sub-populations for all samples.
Gating strategy	Cells were gated by FSC and SSC plots (a representative gating is shown in Figure 8 in the supplementary information).

☒ Tick this box to confirm that a figure exemplifying the gating strategy is provided in the Supplementary Information.
